# *Zoysia japonica* Chlorophyll *b* Reductase Gene *NOL* Participates in Chlorophyll Degradation and Photosynthesis

**DOI:** 10.3389/fpls.2022.906018

**Published:** 2022-05-06

**Authors:** Jin Guan, Ke Teng, Yuesen Yue, Yidi Guo, Lingyun Liu, Shuxia Yin, Liebao Han

**Affiliations:** ^1^College of Grassland Science, Beijing Forestry University, Beijing, China; ^2^Institute of Grassland, Flowers, and Ecology, Beijing Academy of Agriculture and Forestry Sciences, Beijing, China

**Keywords:** chlorophyll *b* reductase, chlorophyll degradation, photosynthesis, chlorophyll fluorescence, *Zoysia japonica*

## Abstract

The degradation of chlorophyll is of great significance to plant growth. The chlorophyll *b* reductase NOL (NYC1-like) is in charge of catalyzing the degradation of chlorophyll *b* and maintaining the stability of the photosystem. However, the molecular mechanisms of NOL-mediated chlorophyll degradation, senescence, and photosynthesis and its functions in other metabolic pathways remain unclear, especially in warm-season turfgrass. In this study, *ZjNOL* was cloned from *Zoysia japonica*. It is highly expressed in senescent leaves. Subcellular localization investigation showed ZjNOL is localized in the chloroplast and the bimolecular fluorescence complementation (BiFC) results proved ZjNOL interacts with ZjNYC1 *in vivo*. *ZjNOL* promoted the accumulation of abscisic acid (ABA) and carbohydrates, and the increase of *SAG14* at the transcriptional level. *ZjNOL* simultaneously led to the excessive accumulation of reactive oxygen species (ROS), the activation of antioxidant enzymes, and the generation of oxidative stress, which in turn accelerated senescence. Chlorophyll fluorescence assay (JIP-test) analysis showed that *ZjNOL* inhibited photosynthetic efficiency mainly through damage to the oxygen-evolving complex. In total, these results suggest that *ZjNOL* promotes chlorophyll degradation and senescence and negatively affects the integrity and functionality of the photosystem. It could be a valuable candidate gene for genome editing to cultivate *Z. japonica* germplasm with prolonged green period and improved photosynthesis efficiency.

## Introduction

Commonly known as a warm-season turfgrass, *Zoysia japonica* (2n = 4x = 40) has many remarkable characteristics, including minimal maintenance, excellent tolerance to drought, salinity, and freezing, good ability to conserve water and soil, and excellent traffic tolerance (Patton and Reicher, 2007; Teng et al., 2017, 2018). Nevertheless, the short green period and unaesthetic appearance during senescence hamper its further popularization and utilization (Teng et al., 2016, 2021b). Therefore, it is critical to interpret the molecular regulation mechanism of chlorophyll degradation and photosynthesis with the help of molecular biology.

In the process of plant senescence and maturation, efficient chlorophyll degradation can lead to rapid chlorosis of plants and the emergence of carotenoids and anthocyanins to form various leaf and flower colors (Hebbar et al., 2014). Chlorophyll degradation is a crucial part of the aging and maturation process, facilitating the transport of nutrients from aging tissues and organs to reproductive and storage organs (Lim et al., 2007; Hebbar et al., 2014; Zhang et al., 2022). The nitrogen in chloroplasts accounts for 75% of the total nitrogen content of the entire photosynthetic system, and 95% of the nitrogen in seeds comes from nitrogen degraded in leaves (Taylor et al., 2010). Chlorophyll degradation removes toxic substances produced during photosynthesis, maintaining cell viability and efficient nutrient redistribution. Chlorophyll degradation is also a prerequisite for the degradation of light-harvesting complexes (LHCs) in senescent leaves, which is crucial for fully utilizing nitrogen in chloroplasts (Hörtensteiner and Feller, 2002). Therefore, the degradation of chlorophyll has significance for the growth of plants and must be strictly regulated.

The chlorophyll of green plants consists of two components, chlorophyll *a* and chlorophyll *b*. Chlorophyll degradation is a complex process that requires the participation of multiple enzymes (Kuai et al., 2018). The degradation of chlorophyll first occurs through the process of converting chlorophyll *b* to chlorophyll *a*, which is the chlorophyll cycle (Shimoda et al., 2012). Non-Yellow Coloring 1 (NYC1) and NYC1-like (NOL) are two key enzymes that catalyze the initial step in chlorophyll *b* degradation to 7-hydroxymethyl-chlorophyll *a* (Shimoda et al., 2012). In many green plants, NYC1 and NOL physically interact and may function as an enzymatic complex to co-catalyze the degradation of chlorophyll *b* (Sato et al., 2010; Sakuraba et al., 2012; Yu et al., 2017; Teng et al., 2021b). In addition, functional differentiation was found between *NOL* and *NYC1* in *Oryza sativar*, *Arabidopsis thaliana*, and *Lolium perenne* (Kusaba et al., 2007; Sato et al., 2010; Yu et al., 2021).

In our previous study, we cloned *ZjNYC1* in *Z. japonica* and found *ZjNYC1* accelerates chlorophyll degradation and leaf senescence (Teng et al., 2021b). *ZjNOL* shows 42.78% amino acid identity to *ZjNYC1*. However, despite the knowledge of NOL’s roles in chlorophyll *b* degradation, the molecular mechanisms underlying NOL-mediated chlorophyll degradation and photosynthesis in *Z. japonica*, and whether its functions differ with *ZjNYC1* are unclear. Leaf senescence induced the expression of *NYC1* and *NOL*, and a close similarity between the phenotypes of *nol* and *nyc1* mutants suggested that *NYC1* and *NOL* have similar functions in leaf senescence (Kusaba et al., 2007; Sato et al., 2010). In Arabidopsis, *NOL* mainly plays a role in the vegetative growth stage and does not significantly promote the leaf senescence process (Sakuraba et al., 2012). These results suggest that the functions of *NYC1* and *NOL* in regulating leaf senescence may be different depending on the plant. However, the mechanisms underlying why the same enzyme performs different functions in mode and non-mode plants remain unknown.

The objectives of this study were to characterize the function and determine the molecular mechanisms of *ZjNOL* in photosynthesis, chlorophyll degradation, and senescence. In addition, clarifying the functional differences in photosynthesis between *ZjNOL* and *ZjNYC1* also was our concern. The information will contribute to the genetic improvement and breeding projects for *Z. japonica* in the future.

## Materials and Methods

### Plant Materials and Growth Conditions

We purchased *Z. japonica* seeds (cv. Zenith) from Patten Seed Company (Lakeland, GA, United States). We sowed them in Klasmann TS1 peat substrate (Klasmann-Deilmann GmbH, Geeste, Germany). Plants were cultivated in climate chambers at 28/25°C (day/night), with a 14-h photoperiod and an average photosynthetic active radiation (PAR) of 400 μmol m^−2^ s^−1^. The plants were watered once a week with Hoagland nutrition solution.

### *ZjNOL* and Its Promoter Cloning

Total RNA was extracted from *Z. japonica* leaves using the Plant RNA Kit (Omega, Georgia, United States). Next, cDNA was generated using the PrimeScriptTM RT reagent Kit (TaKaRa, Dalian, China). We used the CTAB method to obtain genomic DNA. Then, *ZjNOL* and its promoter sequence were amplified using the *Z. japonica* genome database information. The PCR products were purified by the Cycle-Pure Kit (Omega, Georgia, United States) and connected to the pMD-19T cloning vector (TaKaRa, Dalian, China). The plasmids pMD-ZjNOL and pMD-*ZjNOL*pro were obtained after sequencing verification and stored at −80°C.

### Plasmid Construction

The primers used for gene cloning, expression analysis, and plasmid construction in this experiment are listed in Supplementary Table 1. To generate the *ZjNOL*pro::GUS constructs for GUS staining analysis, we inserted the *ZjNOL* promoter sequence into the pCambia1391Z vector. To observe subcellular localization, we constructed the plasmid 3302Y3-ZjNOL, encoding a ZjNOL-YFP fusion protein and driven by a CaMV 35S promoter. The vectors 35S-pSPYCE-YFP and 35S-pSPYNE-YFP were used for bimolecular fluorescence complementation (BiFC). For yeast two-hybrid analysis, we constructed the vectors pGBKT7 and pGADT7. Coding sequences of *ZjNOL* were recombined into the pTA7002 vector to generate *ZjNOL*-overexpressing Arabidopsis lines. The control plants (CK) were using the pTA7002 empty vector.

### Bioinformatic Analysis of *ZjNOL* and Its Promoter Sequence

We performed the BLAST analysis on the NCBI database to search for homologs. The neighbor-joining method was used to construct a phylogenetic tree using the MEGA 11 software (Tamura et al., 2021). To further confirm the evolutionary selection types of these *NOL* genes, we calculated the Ks/Ka ratio using DnaSP6 software (Rozas et al., 2017). The PlantCARE database was used to predict the *cis*-elements in the promoter sequence (Lescot et al., 2002). The compute pI/Mw tool was used to calculate the molecular weight (MW) and theoretical isoelectric point (PI).^1^ ProtComp 9.0^2^ and TargetP 1.1^3^ were used to predict subcellular localization characteristics.

### Quantitative Real-Time PCR

To analyze the expression pattern of the *ZjNOL*, we extracted total RNA from various tissues (roots, stolons, stems, and leaves) and three different development stages (young, mature, and senescent) of leaves. In addition, we treated 3-month-old plants after 12 h induction with hormones, such as 10 μmol GA, 10 μmol methyl jasmonate (MeJA), 10 μmol ABA, and dark. We collected the tissues after induced 0, 0.5, 1, 3, 6, and 12 h. We used four different RNA templates in each study. Each set came with three technical replicates. The qRT-PCR evaluation and data analysis were carried out in accordance with the ΔΔCt method (Teng et al., 2017). *Zoysia japonica β-actin* (GenBank accession no. GU290546) was selected as the housekeeping gene.

### Subcellular Localization and Protein Interaction Analysis

Plasmid 3302Y3-ZjNOL was transformed into *Z. japonica* protoplasts to investigate subcellular localization. The transient gene expression system of *Z. japonica* protoplasts was modified by a previously reported protocol (Yoo et al., 2007). For BiFC analysis, we cloned *ZjNOL* and *ZjNYC1* and fused them with split YFP^N^ and YFP^C^ fragments to co-transform. The yeast two-hybrid experiment, which examined the interaction between ZjNOL and ZjNYC1, was repeated three times according to the instructions of Clontech. We used an SP8 laser confocal scanning microscope for fluorescence observation of protein subcellular localization and BiFC analysis (Leica, Mannheim, Germany).

### Development and Characterization of Transgenic *Arabidopsis thaliana* Lines

Using the floral dip method, *A. tumefaciens* GV3101 transformed with the pTA7002-ZjNOL plasmid was used to generate transgenic *A. thaliana* lines. The harvested seeds were tested on MS medium containing 30 mg/L hygromycin. Following PCR confirmation, only T3 lines with 100% hygromycin resistance were harvested for further morphological analysis. The 3-week-old lines were transferred from MS medium to filter paper soaked in ddH_2_O with 30 M dexamethasone (DEX; Sigma-Aldrich, Munich, Germany) and 0.01% Tween-20. We photographed the seedlings after 4 days of induction.

The chlorophyll content was determined using a previously published protocol (Teng et al., 2016). ELISA kit (H251), H_2_O_2_ assay kit (A064-1-1), and inhibition superoxide anion assay kit (A052-1-1), obtained from Nanjing Jiancheng Bioengineering Institute, Nanjing, China, were used to assay ABA content, H_2_O_2_ content, and inhibit superoxide anion activity, respectively. The plant soluble sugar content test kit (A145-2-1), starch content kit (A148-1-1), and malondialdehyde (MDA) assay kit (MDA-2-Y), purchased from the Jiancheng Bioengineering Institute, Nanjing, China, were used to determine the content of soluble sugars, starch, and MDA. According to the manufacturer’s instructions, we measured superoxide dismutase (SOD), peroxidase (POD), catalase (CAT), and ascorbate peroxidase (APX) activity using reactive oxygen species (ROS) assay kits (Jiancheng Bioengineering Institute, Nanjing, China). All experiments in this study included at least three biological replicates.

To determine chlorophyll fluorescence parameters, we used the Handy PEA analyzer (Hansatech, Kings Lynn, United Kingdom) according to the instructions provided by the manufacturer. After 30 min of dark adaption, leaves were exposed to 2 s saturation light pulses of 3,500 mol photons m^−2^ s^−1^ to measure chlorophyll fluorescence. Ten replicants were used for the CK, line-10, and line-31. We calculated the photosynthetic parameters and the average values of the same group, and a Student *t*-test was used to differentiate the different parameters by comparison to the control. To visualize the data, we used Origin Pro v.2019b (OriginLab Corporation, Northampton, MA, United States).

### Statistical Analysis

We used SPSS version 18.0 (IBM, Chicago, IL, United States) to analyze variance and verify it by a Student *t*-test at a significance level of 0.05 to determine the effect of *ZjNOL*-overexpressing on leaf physiology and transcription level. We used Fisher’s protected least significant difference (LSD) test at the 0.05 probability level to analyze the expression characteristics of *ZjNOL*. All data were presented as means ± SD (*n* ≥ 3).

## Results

### Cloning and Bioinformatic Analysis of *ZjNOL*

**Figure 1:**
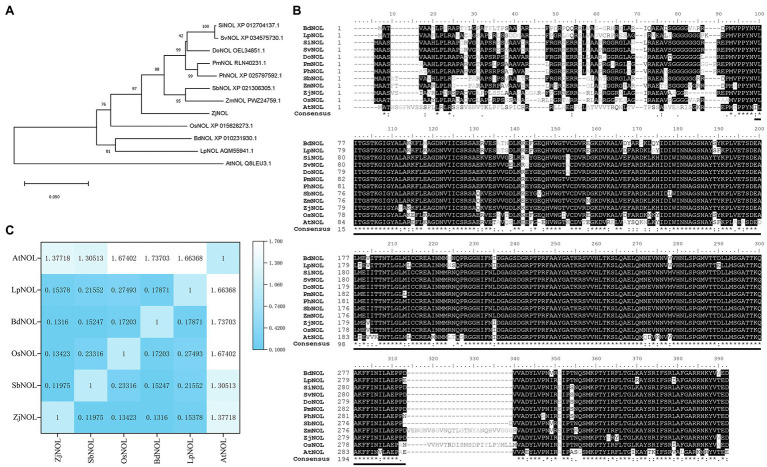
Bioinformatic analysis of *ZjNOL*. **(A)** Phylogenetic tree. The neighbor-joining tree was constructed using MEGA 11, orthologous proteins in *Setaria italica* (SiNOL), *Setaria viridis* (SvNOL), *Dichanthelium oligosanthes* (DoNOL), *Panicum miliaceum* (PmNOL), *Panicum hallii* (PhNOL), *Sorghum bicolor* (SbNOL), *Zea mays* (ZmNOL), *Zoysia japonica* (ZjNOL), O*ryza sativa* (OsNOL), Brachypodium distachyon (BdNOL), *Lolium perenne* (LpNOL), and *Arabidopsis thaliana* (AtNOL) were used for analysis. **(B)** Multiple sequences alignment and conserved domain prediction. **(C)** Calculation of the Ka/Ks.

Using the *Z. japonica* genome and the full-length transcriptome databases as references, we designed the primers and cloned the *ZjNOL* (accession number: OL581613) using RT-PCR. The sequencing results showed that the CDS sequence of the *ZjNOL* was 1,035 bp in length and encoded a total of 344 amino acids. Analysis of homologous sequences revealed that *ZjNOL* contains an SDR domain (Figure 1A), with theoretical isoelectric points and molecular weights of 9.76 and 37.39 kD, respectively. Furthermore, the results of the phylogenetic analysis showed that *ZjNOL* is more closely related to NOL of sorghum (*Sorghum bicolor*; Figure 1B). We calculated the synonymous and non-synonymous substitution rates (Ka/Ks) to further confirm the evolutionary selection types of these *NOL* genes, and the results showed that some *NOL* homologous genes were under diversifying selection (Ka/Ks > 1; Figure 1C).

### Isolation of *ZjNOL* Promoter and GUS Staining Assay

**Figure 2:**
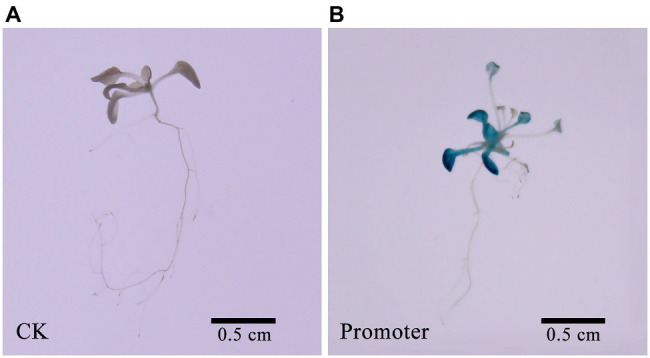
GUS staining results of promoter. **(A)** CK. **(B)** Promoter.

Using *Z. japonica* genomic DNA as a reference, we amplified a 712 bp nucleotide promoter sequence. Furthermore, we predicted and analyzed the *cis*-acting elements of the *ZjNOL* promoter using the PlantCARE website. In addition to the basic functional elements of the promoter, it also contains an ABRE element that responds to ABA induction, five MYB transcription factor binding sites, and three light-responsive elements (Table 1). To further clarify the activity characteristics of the *ZjNOL* promoter, the *ZjNOL*pro::GUS expression vector was constructed and transformed into Arabidopsis. GUS histochemical staining of transgenic seedlings showed that the promoter could drive GUS gene expression in transgenic *Arabidopsis thaliana*, and a large amount of blue was found in the leaves (Figure 2).

**Table 1:** *cis*-elements prediction in the *ZjNOL* promoter.

*cis*-element	Sequences	Amount	Function
ARE	AAACCA	2	Anaerobic inducible
AAGAA-motif	GAAAGAA	1	Unknown
CAAT-box	C(C)A(A)AT	8	Promoter structure
G-Box	CACGTT	1	Light response
MYC	CATG/TTG	5	MYB binding site
GT1-motif	GGTTAA	1	Light response
Sp1	GGGCGG	1	Light response
ABRE	ACGTG	1	ABA response
TATA-box	TATA(A)(A)(A)	19	Promoter core element

### Expression Characters of *ZjNOL*

**Figure 3:**
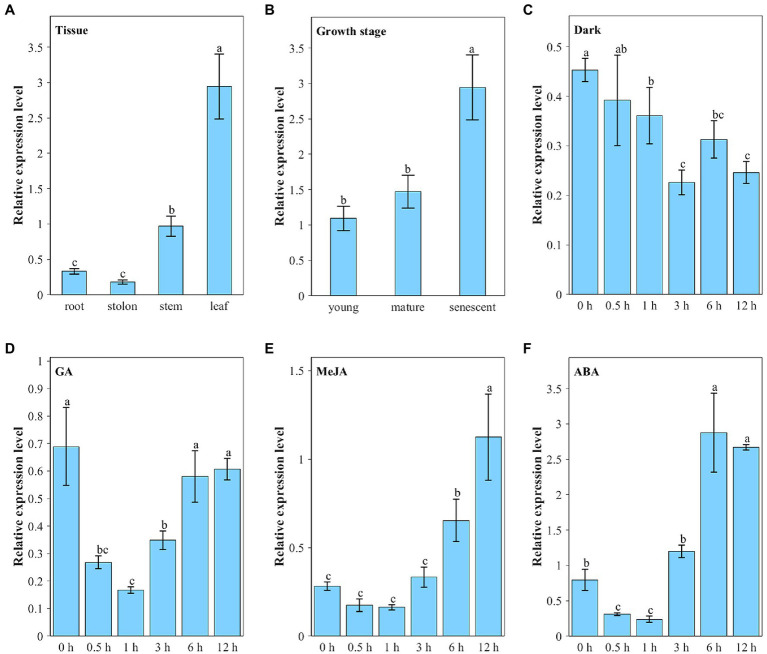
Transcriptional expression characters of *ZjNOL*. **(A)** Different tissues; **(B)** leaves at different growth stages; continuous expression tendency under different treatments: dark **(C)**, GA **(D)**, MeJA **(E)**, and ABA **(F)**. Data were expressed as mean ± SD (*n* = 4). Different letters indicate significant differences at *p* ≤ 0.05 based on Fisher’s protected least significant difference (LSD) test.

We analyzed the expression pattern of the *ZjNOL* gene using qRT-PCR. The results showed that all the tissues could detect the *ZjNOL* gene expression (Figure 3A). Nevertheless, its expression in leaves was significantly higher than that in other tissues (*p* < 0.05), and its expression in stems was also significantly higher than roots and stolons (*p* < 0.05). This result indicates that the expression of the *ZjNOL* is correlated with the content of chlorophyll positively. To determine whether the *ZjNOL* is involved in chlorophyll degradation during leaf senescence, we analyzed the *ZjNOL* gene expression in young, mature, and senescent leaves for qRT-PCR. The results showed that the *ZjNOL* was more actively expressed in senescent leaves, indicating that the *ZjNOL* was involved in the senescence process (Figure 3B).

The *ZjNOL* gene promoter contains *cis*-elements involved in ABA, JA, and light response. Therefore, we analyzed the expression pattern of *ZjNOL* under these treatments. Under dark treatment, the expression of *ZjNOL* showed a slow downward trend (Figure 3C). Ten-micromole GA treatment significantly inhibited the expression of *ZjNOL* within 0–3 h and recovered to the initial level after 6 h (Figure 3D). Ten-micromole MeJA treatment had a significant inducing effect on *ZjNOL*, and the expression level at 12 h was four times higher than that at 0 h (Figure 3E). Ten-micromole ABA treatment showed a trend of first inhibiting and then promoting the expression of *ZjNOL*, and the expression level was the lowest at the 1st hour, 0.4 times that of the 0 h; it reached the highest level at the 6th hour, which was 3.6 times that of the 0 h (Figure 3F).

### Subcellular Localization of ZjNOL

**Figure 4:**
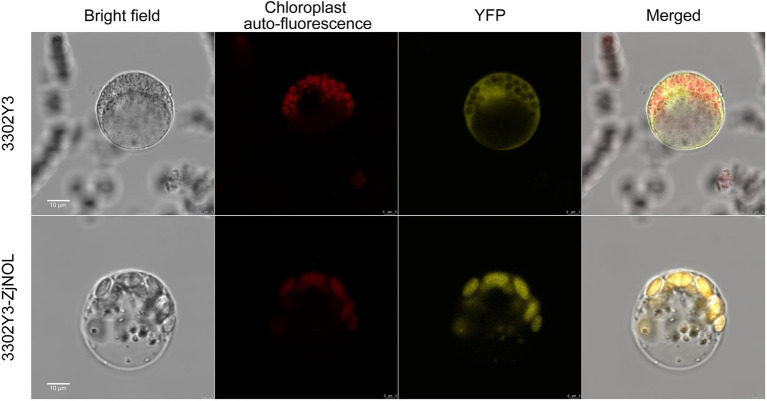
Subcellular localization of ZjNOL in *Zoysia japonica* protoplast. The results showed that ZjNOL was localized in the chloroplast.

**Figure 5:**
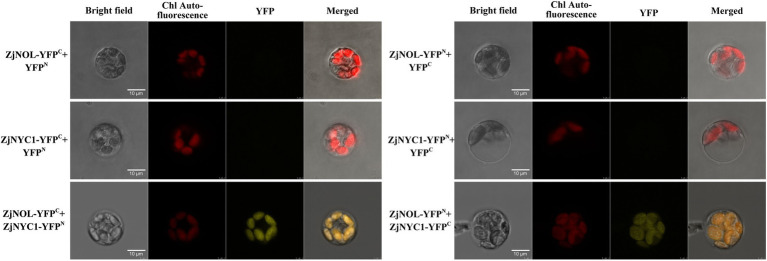
BiFC analysis of the interaction between ZjNYC1 and ZjNOL.

We further used the *Z. japonica* protoplast transient expression system to observe the subcellular localization of ZjNOL, and the results showed that the YFP signal was detected in chloroplasts (Figure 4). Theoretically, NOL can interact with NYC1 in plants such as Arabidopsis, rice, and ryegrass. We performed BiFC analysis on ZjNOL and ZjNYC1 to clarify whether NOL and NYC1 proteins in *Z. japonica* can interact. The results indicate that ZjNOL and ZjNYC1 can interact with each other in *Z. japonica* chloroplasts (Figure 5). In the yeast system, however, ZjNOL and ZjNYC1 did not interact with each other (Supplementary Figure 1).

### Overexpression of *ZjNOL* Accelerated Chlorophyll Degradation and Senescence

**Figure 6:**
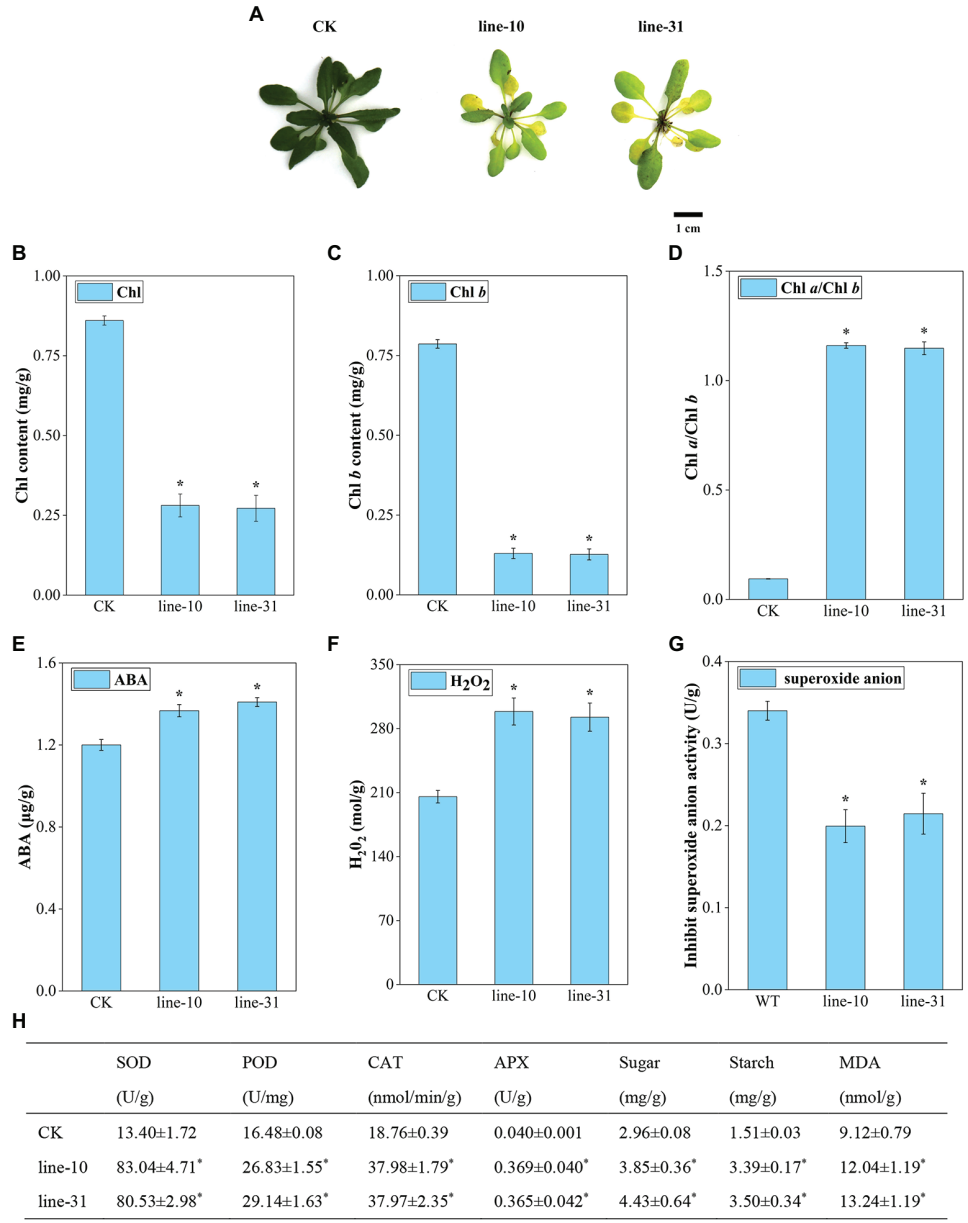
Morphological and physiological assessment of *ZjNOL*-overexpressing lines. **(A)** Photographs of transgenic plants compared with control. **(B)** Chlorophyll (Chl) contents. **(C)** Chlorophyll *b* (Chl *b*) content. **(D)** Chlorophyll *a*/Chlorophyll *b* (Chl *a*/Chl *b*) ratio. **(E)** ABA content. **(F)** H_2_O_2_ content. **(G)** Inhibit superoxide anion activity. **(H)** Physiological changes of enzyme activities (SOD, POD, CAT, and APX) and carbohydrate content (sugar, starch, and MDA). Results are means of three replicates ± SDs. Asterisks indicate significant differences at *p* ≤ 0.05 based on Student *t*-test.

With the inflorescence infection method, we obtained transgenic Arabidopsis lines to determine the function of the *ZjNOL* gene. After 4 days of induction with 30 μM DEX under normal lighting conditions, the control plants remained green, but the transgenic lines turned yellow (Figure 6A). The total chlorophyll content and the chlorophyll *b* levels in line-10 and line-31 were lower than that of CK significantly (*p* < 0.05), and the ratio of chlorophyll *a*/chlorophyll *b* of the transgenic plants was 12 times higher than that of the control plant (*p* < 0.05). The above results indicated that *ZjNOL* catalyzed the conversion of chlorophyll *b* to chlorophyll *a* and promoted chlorophyll degradation (Figures 6B–D). ABA content and H_2_O_2_ content in the transgenic line were significantly higher than those of the control (Figures 6E,F; *p* < 0.05). The inhibition of superoxide anion activity in transgenic lines was decreased compared to the control plant (Figure 6G; *p* < 0.05). In addition, the content of soluble sugar, starch, and MDA was also markedly higher than that of the CK (Figure 6H; *p* < 0.05). The changes in the antioxidant enzyme system of the transgenic lines showed that POD, CAT, APX, and SOD in the transgenic lines were significantly higher than those in the CK (Figure 6H; *p* < 0.05). The above physiological indicators show (Figure 6) that *ZjNOL* promotes chlorophyll degradation and accelerates the senescence process.

### Transcriptional Characteristics Analysis of *ZjNOL*-Overexpressing Lines

**Figure 7:**
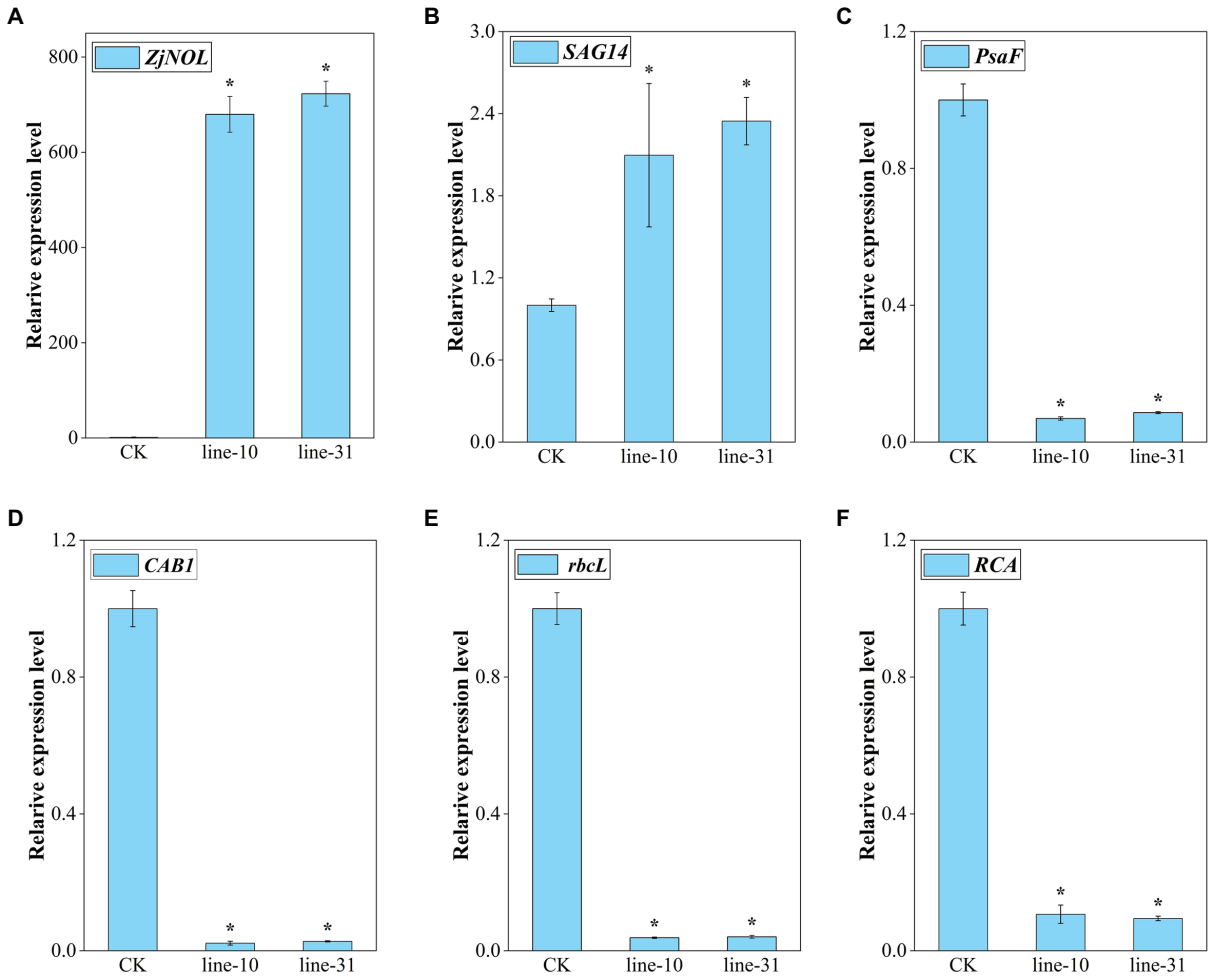
Expression analysis of senescence markers and photosynthesis-related genes. **(A)** *ZjNOL*. **(B)** *SAG14*. **(C)** *PsaF*. **(D)** *CAB1*. **(E)** *rbcL*. **(F)** *RCA*. Data were expressed as mean ± SD (*n* = 4). Asterisks indicate significant differences at *p* ≤ 0.05 based on Student *t*-test.

We analyzed the effect of overexpression of *ZjNOL* in transgenic lines utilizing qRT-PCR. The results showed that *ZjNOL* was expressed efficiently in the transgenic lines (Figure 7A). *SAG14* is a marker gene for senescence. This study found that the overexpression of *ZjNOL* significantly increased the expression of *SAG14*, indicating that it promoted the senescence process of transgenic lines (Figure 7B). Four photosynthetic efficiency marker genes were selected to assess the impact of *ZjNOL* on photosynthesis. The results showed that the expression of *CAB1*, *rbcL*, *RCA*, and *PsaF* significantly downregulated in transgenic lines, reflecting that *ZjNOL* inhibited the photosynthetic efficiency (Figures 7C–F).

### Chlorophyll Fluorescence Induction Curves Analysis of *ZjNOL*-Overexpressing Lines

**Figure 8:**
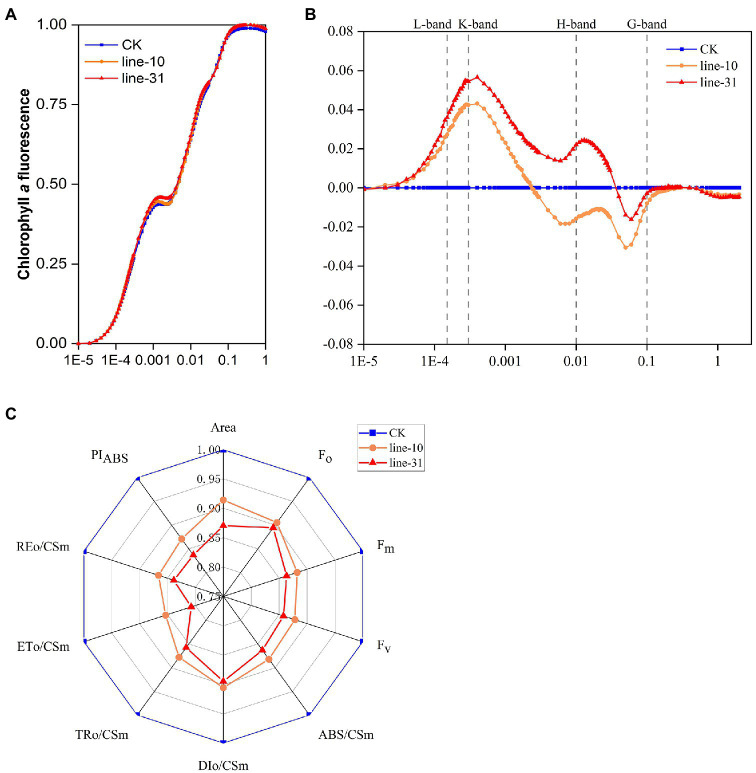
Chlorophyll fluorescence induction curves analysis of *ZjNOL*-overexpressing transgenic lines. **(A)** The standard OJIP curves. **(B)** The relative variable fluorescence V_t_ of the transient chlorophyll *a* fluorescence. **(C)** Radar plot of the fluorescence parameters. The fluorescence parameters were displayed as means of 10 replicates. The K-band and G-band between the transgenic lines and control were significantly different at *p* ≤ 0.05 based on Student *t*-test.

We carried out a standard OJIP analysis of the fluorescence induction curves to evaluate the photosynthetic activity in *ZjNOL* transgenic lines. The results showed that typical O, J, I, and P characteristic steps appeared, as well as an obvious K-step. The expression of *ZjNOL* decreased the minimal fluorescence level (F_o_) and maximal fluorescence level (F_m_), and the maximal variable fluorescence (F_v_) changed little (Figure 8A). We compared the double normalized curves in the O-P transient of chlorophyll fluorescence and found that values of the L-band, K-band were positive, whereas G-band was negative. The positive values of the K-band and the negative values of the G-band in the transgenic lines were significantly higher than those of the control (*p* < 0.05; Figure 8B; Supplementary Figure 2).

The other fluorescence parameters of the JIP-test were further analyzed. We found that some parameters were decreased significantly in the transgenic lines compared to the control and constructed a radar plot to show the detailed parameters (Figure 8C). PI_ABS_ in transgenic lines was significantly lower than that of the control (*p* < 0.05), demonstrating the distributed energy performance index of electron acceptors from the absorption of light energy by PSII to photosynthetic systems (Figure 8C). We drew a phenomenological energy pipeline model to study the influence of *ZjNOL* overexpression (Supplementary Figure 3). It showed that the specific flux membrane model parameters, including ABS/RC, TRo/RC, of the transgenic lines increased, while ETo/RC and DIo/RC did not change much. At the same time, the phenomenological flux leaf model and the radar plot all indicated that parameters of the transgenic lines, including ABS/CSm, TRo/CSm, ETo/CSm, and DIo/CSm, were reduced (*p* < 0.05; Figure 8C; Supplementary Figure 3). In addition, the inactive reaction centers of the transgenic lines increased.

## Discussion

In this study, the full-length *ZjNOL* sequence was cloned. We found that *ZjNOL* was phylogenetically close to its orthologs of *Sorghum bicolor* and shared conserved sequence features as well as similar expression patterns and subcellular localization in chloroplasts. To judge the “purifying selection” and “diversifying selection” in the process of plant evolution, we analysis the synonymous and non-synonymous substitution rates of homologous genes (Gaut et al., 1996). Most chlorophyll catabolism genes, such as *NYC1* and *PPH*, are under purifying selection (Ka/Ks < 1; Teng et al., 2021a,b). However, some *NOL* homologous genes were under diversifying selection (Ka/Ks > 1; Figure 1C). This result is consistent with the reports of perennial ryegrass, indicating that *NOL* has functional diversity in different species (Yu et al., 2021). Therefore, it inspired us to investigate the function of ZjNOL in the typical warm-season turfgrass, *Z. japonica.*

Chlorophyll *b* reductase enzymes, *NYC1* and NYC1-like (*NOL*), play critical roles in the chlorophyll cycle. The BiFC and yeast two-hybrid analysis showed that ZjNOL and ZjNYC1 could interact *in vivo* but not in the Clontech yeast two-hybrid system. This result is consistent with the previous reports in *Z. japonica* (Teng et al., 2021b) but different from rice and Arabidopsis (Sato et al., 2010; Sakuraba et al., 2012). We speculate that there are some differences in the functions of *ZjNOL* and *ZjNYC1* among species. At the same time, there might be some substances in *Z. japonica* that regulate the interaction between ZjNOL and ZjNYC1. SGR is required for protein interactions with the chlorophyll catabolic enzyme (CCE; Sakuraba et al., 2012). Therefore, it is necessary to deeply explore the external factors of the interaction between ZjNOL and ZjNYC1 in future research.

Consistent with the findings in rice (Kusaba et al., 2007), Arabidopsis (Horie et al., 2009), and perennial ryegrass (Yu et al., 2021), the expression of *ZjNOL* was positively correlated with senescence. In *A. thaliana*, overexpression of *AtNOL* resulted in accelerated leaf senescence and chlorophyll *b* degradation in *A. thaliana* (Jia et al., 2015). In *N. benthamian*, the transient overexpression of *LpNOL* accelerated leaf senescence and chlorophyll *b* degradation. Perennial ryegrass *LpNOL* RNA interference (*NOLi*) delays leaf senescence (Yu et al., 2021). In this study, the overexpression of *ZjNOL* accelerated total chlorophyll and chlorophyll *b* degradation, demonstrating that *ZjNOL* is the functional orthologous *NOL* in *Z. japonica*.

The accumulation of ABA plays a positive role in the promotion of senescence (Schippers et al., 2015). *SAG14* is a commonly accepted senescence marker (Wan et al., 2018). Overexpression of *ZjNOL* significantly increased the expression of *SAG14* and the content of ABA, indicating the accelerated senescence process in the *ZjNOL*-overexpressing lines. Carbohydrates, such as soluble sugar and starch, act as energy reserves and are essential for plant growth (Taiz and Zeiger, 2002). Sugar and starch levels are high in senescent Arabidopsis and tobacco leaves (Pourtau et al., 2006; Gan, 2010). Sucrose and starch accumulation can hasten leaf senescence (Lim et al., 2007). The balance of SOD, POD, and APX or CAT activities is crucial for determining superoxide radicals and H_2_O_2_ steady-state levels (Mittler, 2002; Zhang et al., 2021). One of the products of lipid degradation is malondialdehyde (MDA). Thus, increased MDA levels correlated with oxidative stress levels in plants (Bouazizi et al., 2010; Rusinowski et al., 2019). Sugar, starch, and MDA play roles in plant senescence regulation (Wingler et al., 2009; Gan, 2010). In this study, the content of MDA, sucrose, and starch in *ZjNOL*-overexpressing lines was increased significantly higher than in control lines (*p* < 0.05). *ZjNOL* reduced their ability to scavenge H_2_O_2_ and superoxide anion, resulting in excessive accumulation of ROS, activation of antioxidant enzymes, and production of oxidative stress. Consequently, it proved that *ZjNOL* accelerated leaf senescence.

*PsaF*, *CAB1*, *rbcL*, and *RCA* were photosynthetic efficiency markers (Teng et al., 2021a). The expression levels of all four photosynthetic efficiency genes were decreased significantly in this study. Transgenic lines also had lower chlorophyll *b* content and higher chlorophyll *a*/*b* ratio. Overexpression of *ZjNOL* resulted in chlorophyll degradation and destruction of photosynthetic activity in *Z. japonica*. This result is consistent with reports of perennial ryegrass (Yu et al., 2021).

“JIP-test” analysis was widely used to plant gene function studies due to their simplicity, rapidity, ease of reproducibility, and the ability to obtain large amounts of reliable data (Zhang et al., 2020; Bi et al., 2021; Wang et al., 2021). The transient induction curves and JIP-test parameters provide us with information to understand the changes in structural and functional efficiency of photosynthetic apparatus (Baker, 2008; Bussotti et al., 2010; Kalaji et al., 2014; Sitko et al., 2017). The positive K-band was due to slower electron transport from the donor side because of the oxygen-evolving complex (OEC) inactivation and/or faster electron-withdrawal from the acceptor side (Zagorchev et al., 2020). It indicated that *ZjNOL* inhibits the function of PSII by damaging the oxygen-evolving complex. A positive G-band value reveals that the PSI terminal electron acceptor pool is relatively large (Yusuf et al., 2010). We speculated that *ZjNOL* inhibited PSI activity. The negative G-band and significantly lower REo/CSm values support this hypothesis.

The performance index provides comprehensive information on overall photosynthetic efficiency and performance simultaneously (Bussotti et al., 2010). The reduction in the area of the induction curves indicates that the redox state of the electron transport chain or the stoichiometric ratio of the acceptor sides of PSII and PSI has changed (Kalaji et al., 2016). The initial fluorescence (F_o_) represents fluorescence yield when all reaction centers are open or oxidized (Szopiński et al., 2019). The overexpression of *ZjNOL* promoted chlorophyll degradation and led to a decrease in F_o_. ABS/RC and ABS/CSm are two key indicators that indicate the efficiency of the antenna complex (Szopiński et al., 2019). The reduction in the efficiency of the antenna complex further limited photosynthesis. The decrease in ABS/CSm, TRo/CSm, and ETo/CSm was associated with an increase in the density of inactive reactive centers and the ineffectiveness of PSII (Kalaji et al., 2011; Faseela et al., 2019). In detail, the increase in ABS/RC indicated the rise of absorption flux per reaction center. The enhanced TRo/RC reflected the increase of trapped energy flux per reaction center. The above results indicated that the expression of *ZjNOL* inhibited the utilization of light energy in plants. To sum up, we concluded that the effect of *ZjNOL* inhibited photosynthetic efficiency mainly through damage to the oxygen-evolving complex. It is different from the influence of *ZjNYC1* on photosynthesis, which negatively affects the integrity and functionality of PSII, PSI, and the intermedia electron transport chain (Teng et al., 2021b). Taken together, it is reasonable to assume that *ZjNOL* and *ZjNYC1* function differently in photosynthesis.

## Conclusion

**Figure 9:**
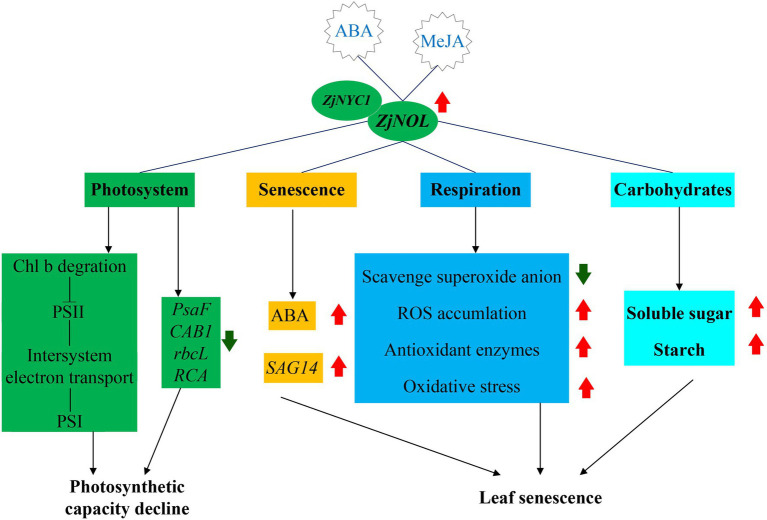
A proposed working model of *ZjNOL* in regulating photosynthesis and senescence.

In conclusion, we proposed a working model of the molecular mechanism of *ZjNOL* in regulating senescence and photosynthesis (Figure 9). *ZjNOL* was highly expressed in senescent leaves. ABA and MeJA induced the expression of *ZjNOL*. ZjNOL is localized in the chloroplast and can interact with ZjNYC1 *in vivo*. *ZjNOL* promoted the accumulation of ABA and carbohydrates and the increase of *SAG14* at the transcriptional level. *ZjNOL* simultaneously led to the excessive accumulation of ROS, the activation of antioxidant enzymes, and the generation of oxidative stress, which in turn accelerated senescence. *ZjNOL* decreased the transcriptional level of photosynthetic efficiency related marker genes and promoted chlorophyll degradation. The JIP-test analysis showed that *ZjNOL* inhibited photosynthetic efficiency mainly through damage to oxygen-evolving complex, which is different from the influence of *ZjNYC1* on photosynthesis. In total, these results suggest that *ZjNOL* promotes chlorophyll degradation and senescence and negatively affects the integrity and functionality of the photosystem. This gene has valuable genetic editing value for cultivating new varieties with stay-green characteristics and improved photosynthesis efficiency.

## Data Availability Statement

The original contributions presented in the study are included in the article/Supplementary Material, and further inquiries can be directed to the corresponding authors.

## Author Contributions

SY and LH conceived the study and designed the experiments. JG performed the experiment, analyzed data, and wrote the manuscript. KT, YY, YG, and LL provided suggestions. All authors contributed to the article and approved the submitted version.

## Funding

This study was supported by the National Natural Science Foundation of China (nos. 31971770 and 31901397), and the Scientific Funds of Beijing Academy of Agriculture and Forestry Sciences (KJCX20210431, CZZJ202210, and KJCX20220103).

## Conflict of Interest

The authors declare that the research was conducted in the absence of any commercial or financial relationships that could be construed as a potential conflict of interest.

## Publisher’s Note

All claims expressed in this article are solely those of the authors and do not necessarily represent those of their affiliated organizations, or those of the publisher, the editors and the reviewers. Any product that may be evaluated in this article, or claim that may be made by its manufacturer, is not guaranteed or endorsed by the publisher.

## Footnotes

^1^ https://web.expasy.org/computepi/

^2^ http://www.softberry.com

^3^ http://www.cbs.dtu.dk/services/TargetP-1.1/index.php

## Supplementary Material

Supplementary Figure 1 Yeast two-hybrid analysis of ZjNOL and ZjNYC1.

Supplementary Figure 2 The OJIP curves, K-band, and G-band. **(A)** The OJIP curves. **(B)** K-band. **(C)** G-band.

Supplementary Figure 3 The proportion of phenomenological energy flux parameters of the transgenic lines and the control displayed by the leaf pipeline model. The fluorescence parameters were displayed as means of ten replicates. The width of the arrow represents the relative values of the associated parameters; dark circle represents non-active reaction centers.
