# Supplementary figures and images for: Zoysia japonica Chlorophyll b Reductase Gene NOL Participates in Chlorophyll Degradation and Photosynthesis
Source: Front Plant Sci. 2022 May 6;13:906018. doi: 10.3389/fpls.2022.906018 (PMC9121134; doi:10.3389/fpls.2022.906018)

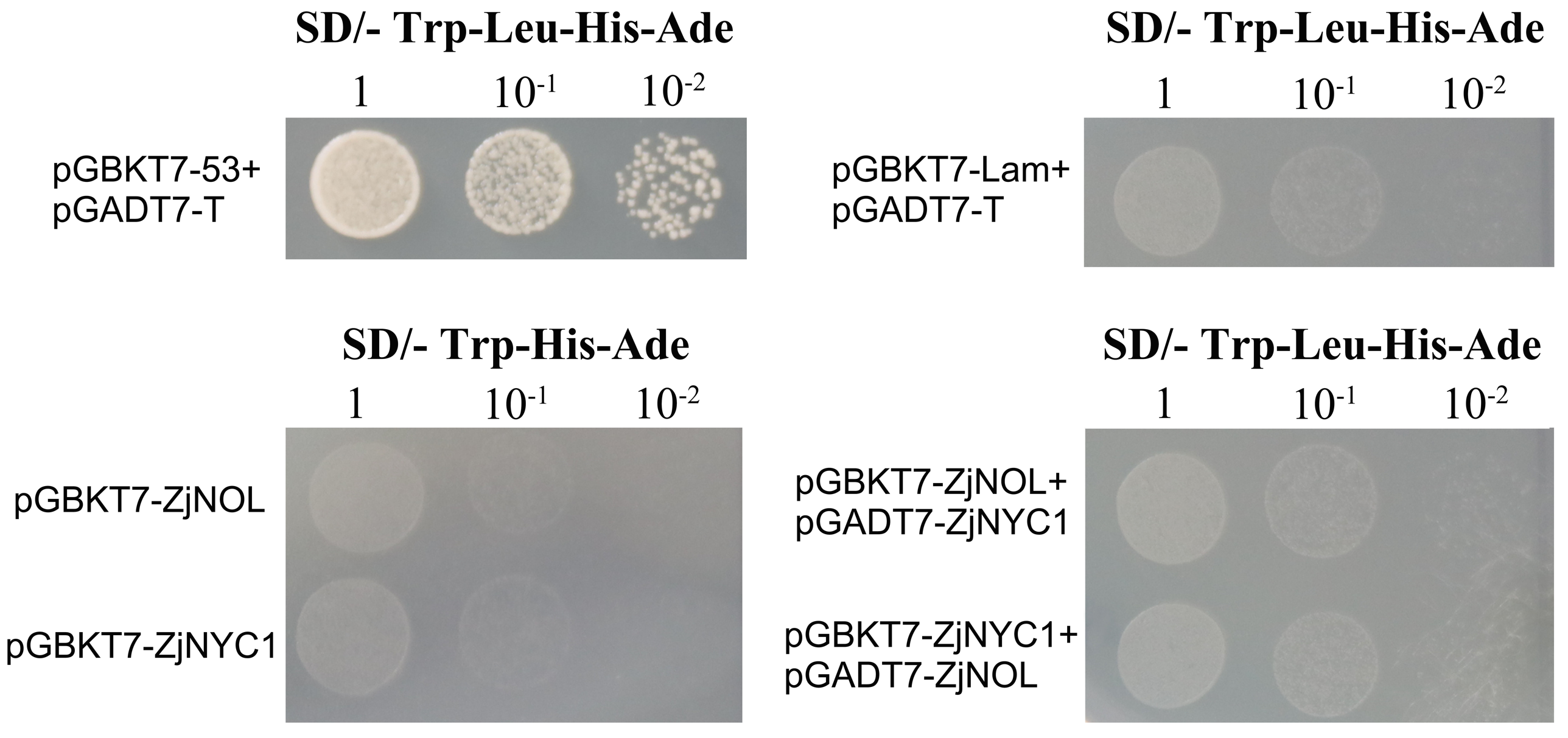

Supplement: Supplementary Figure 1 — Yeast two-hybrid analysis of ZjNOL and ZjNYC1. [file Image_1.JPEG]

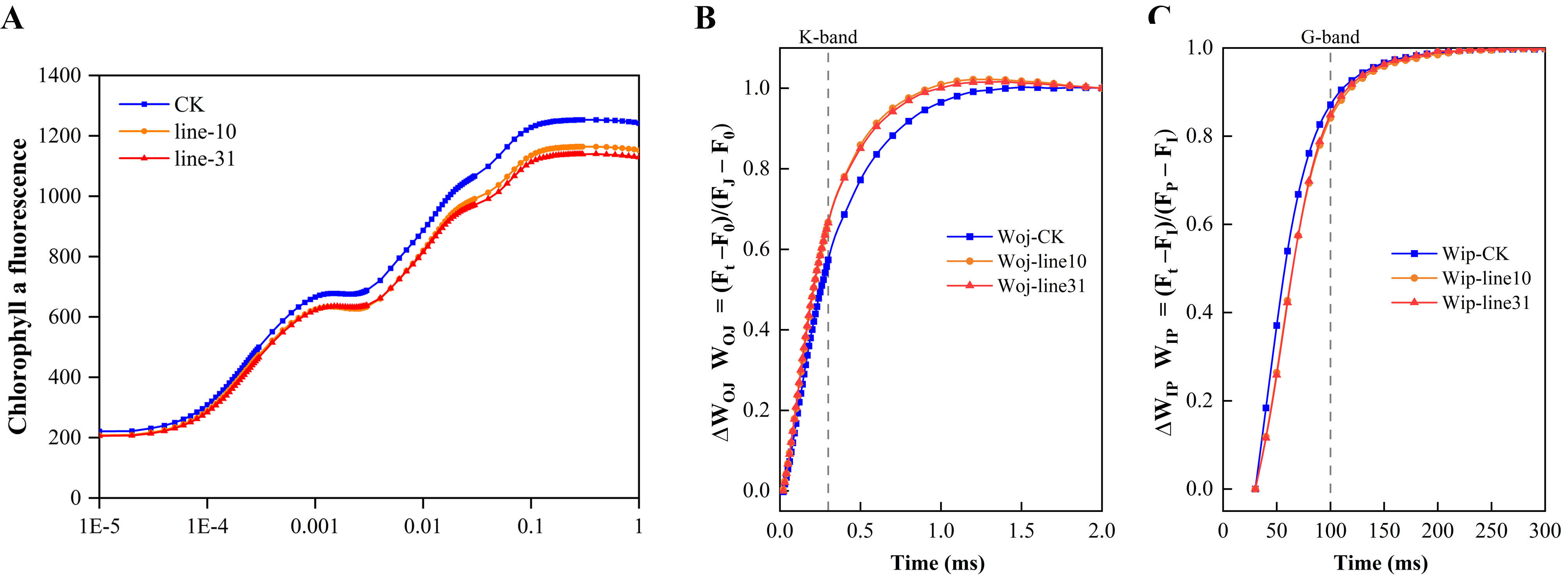

Supplement: Supplementary Figure 2 — The OJIP curves, K-band, and G-band. (A) The OJIP curves. (B) K-band. (C) G-band. [file Image_2.JPEG]
